# Association Between Blood Lead Level With High Blood Pressure in US (NHANES 1999–2018)

**DOI:** 10.3389/fpubh.2022.836357

**Published:** 2022-04-25

**Authors:** Ziyao Huang

**Affiliations:** Department of Cardiovascular Medicine, The Third Affiliated Hospital of Guangzhou Medical University, Guangzhou, China

**Keywords:** blood lead level (BLL), blood pressure, hypertension, NHANES (National Health and Nutrition Examination Survey), high blood pressure (HBP)

## Abstract

**Background:**

Lead is a toxic metal for human health, but the effect on blood pressure (BP) is still controversial. The object of this study was to demonstrate the association between blood lead levels with BP and hypertension (HTN).

**Methods:**

We used the database from the National Health and Nutrition Examination Survey (NHANES, 1999–2018) to perform a cross-sectional study. We performed multivariate regressions to examine the association between blood lead level with HTN and BP, and then a subgroup analysis was performed.

**Results:**

A total of 32,289 participants were included in this study, but no significant difference was found between blood lead levels and HTN. However, the association between blood lead levels with systolic and diastolic pressure became positive. In the subgroup analysis stratified by race, the association between non-Hispanic white and black people still existed.

**Conclusion:**

The association between blood lead levels with HTN was not significant, but it was positively associated with BP. Besides, the association between non-Hispanic white and black people was also significant.

## Introduction

Lead is a kind of heavy metal that can cause environmental pollution, and it is also a toxic metal that is harmful to human health. For its specific physical and chemical properties, lead is widely used in industry and our lives, such as in mining, batteries, paints, and lead gasoline (1–3). Especially in low and middle-income countries, informal recycling of metal from waste is widespread, which causes more exposure, disease, and death (4, 5).

Lead has occurred in Earth's crust because lead is non-biodegradable, it could persist in the environment for a long time. Lead has two natural types, organic and inorganic forms. Ingestion and inhalation are the most common ways of exposure. Inorganic lead can cross the blood-brain barrier to cause acute lead poisoning, and it is directly absorbed, distributed, and excreted. Organic lead is usually absorbed *via* the skin and respiratory systems and can cross an adult's blood-brain barrier. The absorption rate is about 10–15%, which depends on the lead's physical and chemical features and the condition of an exposed person. In pregnant women and children, the rate may increase to 50% (6). Until 2012, when the blood lead level was 10 or more micrograms per deciliter, it was identified as having a blood lead “level of concern.” Based on the NHANES data from 2007 to 2010, the blood lead reference value (BLRV) for children corresponding to 97.5% was established to be 5 μg/dl. The value above 3.5 μg/dl was defined as a blood lead “reference value” after 14 May 2021 (7). However, no evidence of harmful effects threshold has been established.

Lead can damage the cardiovascular, neurological, renal, reproductive, skeletal, immune, hematological, digestive, and endocrine systems (1, 8–14). In some studies, blood lead levels were associated with high blood pressure (BP) (15–18). However, in previous studies based on the database of NHANES III and NHANES 2003–2010, no significant evidence of blood lead level with hypertension (HTN) was demonstrated (19, 20). However, the association between blood lead levels and BP is uncertain.

## Methods

### Study Population

**Figure 1:**
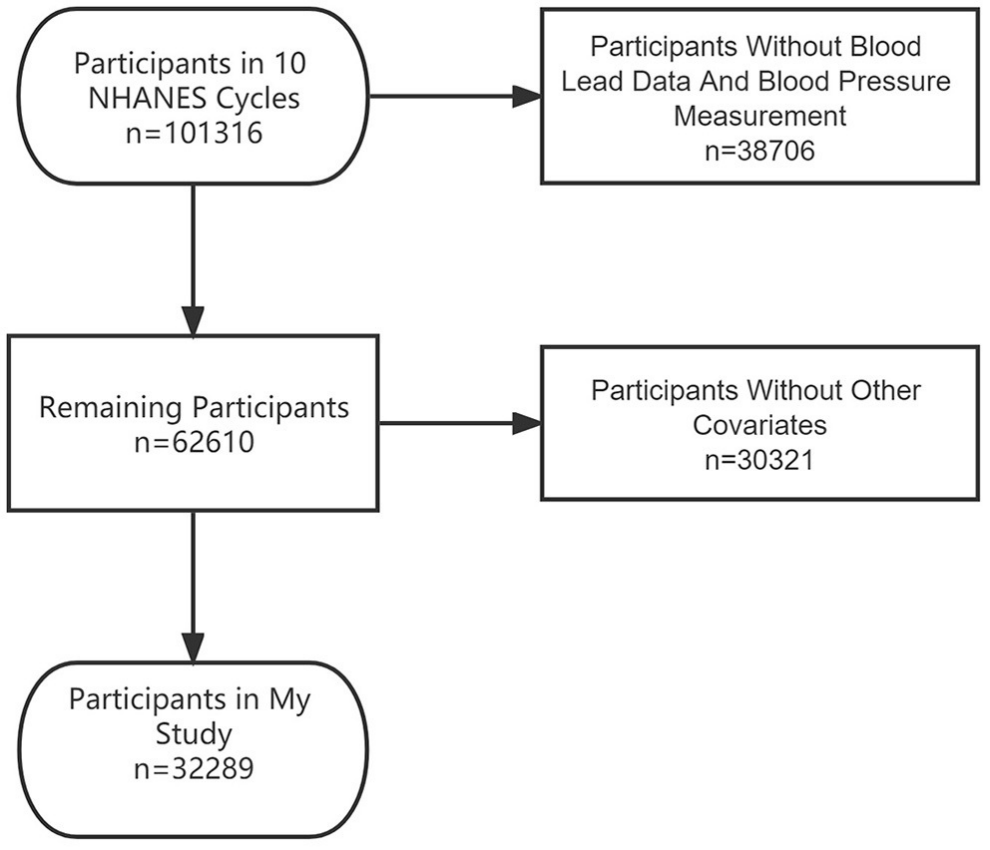
The flowchart of participants.

All the databases could be obtained from the NHANES website (https://wwwn.cdc.gov/nchs/nhanes/Default.aspx), which was a nationally representative survey of nutrition and health condition in the United States. We analyzed the data from the last 10 cycles (1999–2018). A total of 101,316 subjects were included in 10 consecutive NHANES survey cycles covering the periods 1999–2018. There were 38,706 participants without blood lead data or BP measurement excluded in this study. Then, the participants who were aged below 20 or had no data on education level, rate of family income to poverty, body mass index (BMI), alcohol use, and smoking behavior were also excluded, and the final study population of this study was 32,289. The process of recruiting is shown in Figure 1.

### Study Variable

Venous blood was obtained to measure the blood lead level. Blood samples were simply diluted (21) and stored at −20°C before measurement. The measurement of blood lead level was conducted using an Inductively Coupled Plasma Dynamic Reaction Cell Mass Spectrometer (ELAN DRC II, PerkinElmer, Norwalk) in the central laboratory according to the standard protocol (22). The lower limits of detection were 0.3 μg/dl in 1999–2002, 0.28 μg/dl in 2003–2004, 0.25 μg/dL in 2003–2012, 0.07 μg/dl in 2013–2016, and 0.05 μg/dl in 2017–2018. All blood lead levels lower than the lower limit of detection were replaced by the lower limit of detection divided by √2 (23).

Three consecutive BP readings are obtained after resting quietly in a seated position for 5 min and after the participant's maximum inflation level (MIL) has been determined. If a BP measurement is interrupted or incomplete, a fourth attempt may be made. All BP determinations (systolic and diastolic) are taken at the mobile examination center (MEC). Participants with any of the following on both arms are excluded from the exam: rashes, gauze dressings, casts, edema, paralysis, tubes, open sores or wounds, withered arms, a-v shunts, and radical mastectomy. BP measurements are taken in the right arm unless specific conditions prohibit the use of the right arm or if participants report any reason that the BP measurements should not be taken in the right arm (22).

The participants had 1–4 BP readings in the study and anyone without any BP readings was excluded. If they had only one BP reading, then, it was the final record. When they had more than one BP reading, the first reading was always excluded. The BP record was the average of the readings after excluding the first reading.

Hypertension was defined when the participants self-reported HTN or used medication for HTN. For those who did not have self-reported, according to the American Heart Association/American College of Cardiology (AHA/ACC) 2017 guideline for monitoring and diagnosis of HTN. Participants with systolic BP ≥ 130 mmHg or diastolic BP ≥80 mmHg were also considered hypertensive (24).

The details of other covariate data, such as education level, rate of family income to poverty, BMI, alcohol use, and smoking behavior, can be obtained at www.cdc.gov/nchs/nhanes/.

### Statistical Analyses

All the analyses were performed with R (version 3.4.3, http://www.R-project.org) and EmpowerStats software (http://www.empowerstats.com). Continuous variables were reported as mean ± standard deviation (SD), while the categorical variables were percentages. Baseline characteristics were analyzed by a linear regression model for continuous variables and a chi-square test for categorical variables, respectively. We divided the blood lead concentration into four categories. Multivariable logistic regression models were performed to explore the independent association of blood lead concentration with high BP after adjusting for potential confounding factors. Subgroup analyses stratified by sex, race, and BMI were performed. Three models were built: an unadjusted model, a minimally adjusted model (adjusted for age, sex, and race), and a fully adjusted model (adjusted for age, sex, race, education level, rate of family income to poverty, BMI, alcohol use, and smoking behavior). The value of *p* < 0.05 was considered statistically significant.

## Results

### Baseline Characteristic

The characteristics of participants were shown in Table 1, and a total of 32,289 participants aged 20–85 years who had measured data for blood lead and BP were included in this study. There were 54.44% of the participants with HTN, 49.24% were men, and the mean age was 49.68 ± 18.04 years old. In different groups of HTN, prevalence rate, sex, age, race, education level, rate of family income to poverty, BMI, systolic BP, diastolic BP, alcohol use, and smoking behavior are significantly different.

**Table 1:** The characteristics of participants.

	**Mean±SD**	**Q1**	**Q2**	**Q3**	**Q4**	***P* Value**
N	32,289	7,978	7,850	8,303	8,158	
Hypertension (%)	54.44	38.86	52.92	59.16	66.35	<0.001
Age (years)	49.68 ± 18.04	38.39 ± 15.10	48.04 ± 17.26	53.59 ± 16.81	58.32 ± 16.54	<0.001
Male (%)	49.24	31.51	44.48	53.96	66.36	<0.001
Race (%)						<0.001
Mexican American	17.06	16.27	15.76	17.17	18.95	
Other Hispanic	7.84	10.30	9.22	6.90	5.05	
Non-Hispanic White	46.72	45.69	46.00	48.33	46.80	
Non-Hispanic Black	19.98	18.76	19.48	19.05	22.60	
Other Race	8.40	8.97	9.54	8.54	6.59	
Education level (%)						<0.001
< High school	25.67	15.72	22.25	27.29	37.04	
High school	23.31	21.53	22.96	23.85	24.83	
>High school	51.02	62.75	54.79	48.86	38.12	
Ratio of family income to poverty	2.56 ± 1.62	2.61 ± 1.63	2.66 ± 1.64	2.63 ± 1.63	2.34 ± 1.56	<0.001
Alcohol use (%)	86.59	85.03	85.22	86.79	89.21	<0.001
Smoking behavior (%)						<0.001
Never smoke	53.67	69.97	58.54	48.20	38.64	
Still smoke	20.84	12.30	18.36	22.96	29.44	
Quit smoke	25.48	17.74	23.11	28.84	31.92	
Body mass index (kg/m^2^)	29.04 ± 6.74	30.21 ± 7.93	29.41 ± 6.84	28.76 ± 6.28	27.81 ± 5.50	<0.001
Systolic blood pressure (mmHg)	124.25 ± 19.17	117.86 ± 15.90	122.87 ± 17.94	126.16 ± 19.42	129.89 ± 20.88	<0.001
Diastolic blood pressure (mmHg)	70.05 ± 13.47	69.32 ± 12.26	69.97 ± 13.02	70.50 ± 13.75	70.39 ± 14.65	<0.001
Blood lead level (μg/dL)	1.73 ± 1.71	0.57 ± 0.16	1.04 ± 0.14	1.63 ± 0.23	3.63 ± 2.48	<0.001

*Mean±SD for continuous variables: P value was calculated by linear regression model; % for Categorical variables: P value was calculated by chi-square test*.

### The Association of Blood Lead Concentration With High BP

We have used three multivariate logistic regression models to show the relationship between blood lead concentration with high BP in Table 2: model 1, no covariate was adjusted; model 2, age, sex, and race were adjusted; model 3, age, sex, race, education level, rate of family income to poverty, BMI, alcohol use, and smoking behavior were adjusted. We found a significantly positive association between blood lead concentration with high BP in the unadjusted model (Model 1). However, after adjusting by covariates, no significant associations were found for these models (models 2 and 3). In the fully-adjusted model, compared with the lowest blood lead concentration category, the HTN prevalence rate increased (1.00–1.06, *p* = 0.0280). Compared with the lowest quartile group of blood lead levels, the prevalence of HTN in the highest quartiles group increased by 12%.

**Table 2:** Association between blood lead levels (μg/dl) and hypertension.

	**Model 1**	**Model 2**	**Model 3**
	**β (95% CI)**	**β (95% CI)**	**β (95% CI)**
Per 1 μg/dL BLL increase	1.23 (1.21, 1.25)***	0.99 (0.97, 1.00)	1.01 (0.99, 1.03)
BLL (Quartile)			
Q1	Reference	Reference	Reference
Q2	1.77 (1.66, 1.88)***	1.00 (0.93, 1.07)	1.10 (1.02, 1.18)*
Q3	2.28 (2.14, 2.43)***	0.91 (0.85, 0.98)*	1.06 (0.98, 1.14)
Q4	3.10 (2.91, 3.31)***	0.91 (0.84, 0.98)*	1.12 (1.03, 1.22)**
P for trend	1.44 (1.41, 1.47)***	0.96 (0.94, 0.99)**	1.03 (1.00, 1.06)*
Stratified by sex^a^			
Women	1.56 (1.50, 1.62)***	0.99 (0.95, 1.02)	1.03 (0.99, 1.07)
Men	1.09 (1.07, 1.11)***	0.99 (0.97, 1.00)	1.01 (0.99, 1.03)
Stratified by race^b^			
Mexican American	1.05 (1.02, 1.08)***	0.96 (0.93, 0.99)*	0.99 (0.96, 1.02)
Other hispanic	1.16 (1.08, 1.25)***	1.00 (0.95, 1.05)	1.01 (0.95, 1.06)
Non-hispanic white	1.35 (1.31, 1.39)***	0.99 (0.97, 1.02)	1.03 (1.00, 1.06)*
Non-hispanic black	1.36 (1.30, 1.43)***	0.99 (0.96, 1.03)	1.02 (0.98, 1.06)
Other race	1.22 (1.14, 1.30)***	0.99 (0.93, 1.05)	1.04 (0.97, 1.11)
Stratified by BMI^c^			
BMI≥30	1.26 (1.21, 1.30)***	0.99 (0.96, 1.01)	1.00 (0.97, 1.03)
25 < BMI ≤ 30	1.23 (1.20, 1.27)***	1.01 (0.99, 1.04)	1.00 (0.98, 1.03)
BMI ≤ 25	1.35 (1.31, 1.39)***	1.04 (1.01, 1.07)**	1.03 (1.00, 1.06)*

*Model 1: no covariates were adjusted*.

*Model 2: age, sex, race were adjusted.*

*Model 3: age, sex, race, education level, rate of family income to poverty, body mass index (BMI), alcohol use, and smoking behavior were adjusted.*

*^a^All models were not adjusted by sex, ^b^ all models were not adjusted by race, ^c^ all models were not adjusted by BMI.*

*BLL, Blood lead levels; BMI Body mass index.*

^*^p <0.05, ^**^p <0.01, ^***^p <0.001.

There was no significant association in the subgroup analysis stratified by sex. However, in the fully adjusted model, the positive association was only existed in non-Hispanic whites (1.00–1.06, *p* = 0.0380), while the other races did not have a significant association with blood lead concentration. Besides, we could find a positive association when the BMI was not above 25 in the subgroup analysis stratified by BMI level (1.00–1.06, *p* = 0.0320). Furthermore, we tried to explore the association of high BP with blood lead level stratified by sex within the blood lead quartered group, and no significant difference was found in all the full-adjusted models.

The association between blood lead level and systolic and diastolic pressure (Tables 3, 4) was significantly different (0.19–0.42, *p* < 0.0001; 0.14–0.32, *p* < 0.0001, respectively). In the subgroup analysis stratified by sex and race, after fully adjusting, the significant difference only existed in non-Hispanic white or black.

**Table 3:** Association between blood lead levels (μg/dl) and systolic pressure, stratified by sex and race.

	**Model 1**	**Model 2**	**Model 3**
	**β (95% CI)**	**β (95% CI)**	**β (95% CI)**
Per 1 μg/dL BLL increase	1.77 (1.65, 1.89)***	0.26 (0.14, 0.37)***	0.30 (0.19, 0.42)***
Stratified by sex and race			
Men			
Mexican American	0.35 (0.09, 0.60)**	0.05 (−0.18, 0.28)	0.10 (−0.13, 0.34)
Other hispanic	0.36 (−0.04, 0.76)	0.06 (−0.32, 0.43)	0.07 (−0.31, 0.45)
Non-hispanic white	1.23 (1.01, 1.45)***	0.39 (0.18, 0.60)***	0.44 (0.22, 0.66)***
Non-hispanic black	1.21 (0.93, 1.50)***	0.36 (0.08, 0.65)*	0.37 (0.07, 0.67)*
Other Race	1.01 (0.45, 1.56)***	0.24 (−0.28, 0.76)	0.49 (−0.04, 1.03)
Women			
Mexican American	1.67 (1.16, 2.18)***	0.11 (−0.30, 0.53)	0.14 (−0.28, 0.57)
Other hispanic	4.04 (2.89, 5.19)***	0.97 (−0.01, 1.95)	0.84 (−0.15, 1.83)
Non-hispanic white	5.24 (4.81, 5.67)***	0.25 (−0.15, 0.66)	0.63 (0.22, 1.04)**
Non-hispanic black	3.71 (3.19, 4.23)***	0.96 (0.46, 1.46)***	0.99 (0.48, 1.50)***
Other race	3.27 (2.31, 4.24)***	0.25 (−0.60, 1.10)	0.49 (−0.35, 1.34)

*Model 1: no covariates were adjusted*.

*Model 2: age were adjusted.*

*Model 3: age, education level, rate of family income to poverty, BMI, alcohol use, and smoking behavior were adjusted.*

*BLL, Blood lead levels.*

^*^p <0.05, ^**^p <0.01, ^***^p <0.001.

**Table 4:** Association between blood lead levels (μg/dl) and diastolic pressure, stratified by sex and race.

	**Model 1**	**Model 2**	**Model 3**
	**β (95% CI)**	**β (95% CI)**	**β (95% CI)**
Per 1 μg/dL BLL increase	0.15 (0.07, 0.24)***	0.08 (-0.01, 0.17)	0.23 (0.14, 0.32)***
Stratified by sex and race			
Men			
Mexican American	−0.08 (−0.26, 0.11)	−0.10 (−0.28, 0.09)	0.08 (−0.11, 0.26)
Other hispanic	−0.23 (−0.53, 0.08)	−0.27 (−0.58, 0.03)	−0.20 (−0.51, 0.11)
Non-hispanic white	−0.22 (−0.39, −0.05)*	0.18 (0.01, 0.36)*	0.40 (0.22, 0.58)***
Non-hispanic black	0.13 (−0.10, 0.36)	0.09 (−0.15, 0.34)	0.26 (0.00, 0.51)*
Other race	−0.34 (−0.75, 0.06)	−0.23 (−0.64, 0.19)	0.05 (−0.37, 0.48)
Women			
Mexican American	0.06 (−0.26, 0.38)	−0.08 (−0.40, 0.24)	0.08 (−0.25, 0.40)
Other hispanic	0.50 (−0.18, 1.19)	0.36 (−0.35, 1.07)	0.42 (−0.30, 1.14)
Non-hispanic white	−0.16 (−0.45, 0.13)	0.48 (0.15, 0.80)**	0.74 (0.41, 1.07)***
Non-hispanic black	0.46 (0.11, 0.82)*	0.61 (0.22, 0.99)**	0.80 (0.40, 1.20)***
Other race	0.19 (−0.40, 0.79)	−0.03 (−0.65, 0.59)	0.16 (−0.47, 0.79)

*Model 1: no covariates were adjusted*.

*Model 2: age were adjusted.*

*Model 3: age, education level, rate of family income to poverty, BMI, alcohol use, and smoking behavior were adjusted*.

*BLL, Blood lead levels.*

^*^p <0.05, ^**^p <0.01, ^***^p <0.001.

## Discussion

In previous studies, the relationship between blood lead levels and BP was not clear. The studies from the United States (25), Korea (26), and Brazil (27) demonstrated that blood lead level is related to high BP. A Korean study by Kim was used to assess the association between low blood lead levels with BP and HTN in lead-exposed male workers. When the blood lead level was above 6.87 μg/dl, it was associated with HTN. As a result, even in workers with low blood lead levels (<10 μg/dl), BP management appeared to be necessary (17). However, a study using the database of NHANES 2003–2010 showed that the blood lead level had no significant relationship with HTN, which was associated with higher systolic and diastolic pressure levels (20). The study aimed to assess the prevalence of HTN in United States adults and determine the difference between the JNC7 and ACC/AHA 2017 guidelines. They found that, based on JNC7, the overall prevalence of HTN was 31.7%, and the corresponding prevalence was 45.6% when using the new guideline of ACC/AHA (28). In this study, the prevalence of HTN was 54%, which is about 8% more than in Rana's study. First, they defined HTN by BP or using anti-HTN medicines, so they might lose those who answered ‘Ever told you had high BP.' Second, for further analysis, we dropped the data points that did not have the covariates, so it may cause the difference in the prevalence of HTN. Our study used the database from the 10 cycles of NHANES, which had a larger size of population than other studies. In the meantime, the definition of HTN was based on AHA/ACC 2017 guideline, causing a more high BP population than in previous studies.

In race stratified analysis, we found a positive association between blood lead level and HTN only in the full-adjusted model of non-Hispanic whites, while other subgroups had no significant association. Due to reduced statistical power, these associations did not reach statistical significance. Other studies found that the association between blood lead levels and BP was not consistent across races (20, 25). In the meantime, both systolic pressure and diastolic pressure were significantly associated with blood lead levels in non-Hispanic white and black participants. Another study demonstrated that the prevalence of HTN in white, black, and Hispanic people is 46.0, 54.7, and 38.5%, respectively (28). Thus, the race is an important factor for BP, and more evidence should be explored in future studies.

In the BMI stratified analysis, a significant association only existed in the BMI <25 subgroup. There are no studies to explain the reason for this phenomenon. The participants with lower BMI might be less tolerant to lead exposure because of their thinner bodies.

Some studies reported that bone lead is a more ideal marker to explain lead levels in the human body. A half-life of blood lead is about a month, while bone lead is more than 10 years old (29). Thus, the lead in our bodies is mainly reserved in the bones (30). It was difficult to correct the effect of aging using bone lead as a marker because bone level changes as people age. Furthermore, it is measured indirectly using X-ray fluorescence (31) and NHANES did not measure this subject.

This study did not show evidence for blood lead levels causing HTN, however, a growing blood lead level made higher systolic and diastolic pressure, even the increase was about 1 mmHg. However, some analyses showed that an increase of 2 mmHg in systolic pressure added to the risk of stroke (32). Moreover, lead exposure was a family or regional event instead of an individual problem. Therefore, a decrease in systolic or diastolic pressure might affect a lot of people at a population level, and we should pay attention to the harmful effect of lead.

However, the mechanism of lead toxicity for humans was not clear, and free radical damage might be the most possible reason (33). On the other hand, lead might be used instead of calcium because it is also a divalent cation in many signaling pathways level, which affects vascular resistance and increases BP (34). Besides, lead could reduce nitrogen monoxide and guanylate cyclase production in blood vessels, which remodels the vessel and inhibits vascular relaxation (16). More studies are needed to explore the possible mechanisms to find new targets that can help us to understand the molecular mechanisms of lead toxicity.

### Limitation

On one hand, this study demonstrated the association of blood lead level with systolic and diastolic pressure, but not blood lead level with HTN. We should include more participants in the future to increase the population. On the other hand, this study was cross-sectional research, and the data came from an observational survey. It cannot demonstrate the causation but only the association. In the meantime, because of a lack of data covariates, more than half participants were excluded, which might cause bias. Furthermore, BP was affected by many genetic and environmental factors. The information that was not collected in the survey could not be analyzed in the study. The anti-HTN medicine was also an important variable, but most of the participants in the study lacked this data. Further and prospective studies should be completed in the future.

## Conclusion

Increasing blood lead levels are associated with higher systolic and diastolic pressure, but not with HTN. The association between non-Hispanic white and black people is especially significant. As a result, lowering blood lead levels may benefit the general population.

## Data Availability Statement

Publicly available datasets were analyzed in this study. This data can be found here: https://www.cdc.gov/nchs/nhanes/index.htm.

## Ethics Statement

The studies involving human participants were reviewed and approved by NCHS Research Ethics Review Board (ERB). Written informed consent for participation was not required for this study in accordance with the national legislation and the institutional requirements.

## Author Contributions

The author confirms being the sole contributor of this work and has approved it for publication.

## Conflict of Interest

The author declares that the research was conducted in the absence of any commercial or financial relationships that could be construed as a potential conflict of interest.

## Publisher's Note

All claims expressed in this article are solely those of the authors and do not necessarily represent those of their affiliated organizations, or those of the publisher, the editors and the reviewers. Any product that may be evaluated in this article, or claim that may be made by its manufacturer, is not guaranteed or endorsed by the publisher.
